# A retrospective towards a biodegradable material concept for future Indonesian sustainable architecture

**DOI:** 10.1186/s40410-021-00142-1

**Published:** 2021-10-09

**Authors:** Fermanto Lianto, Denny Husin, Clinton Thedyardi, Mieke Choandi, Rudy Trisno

**Affiliations:** 1 Department of Architecture, Universitas Tarumanagara, Jl. S. Parman No. 1, Jakarta, 11440 Indonesia; 2 Projective Cities, The Architectural Association School of Architecture, London, UK

**Keywords:** Architecture, Biodegradable, Concept, Material, Sustainable

## Abstract

The awareness of the negative effect of the intensive usage of synthetic material has led to a significant phenomenon in recent global development. Moving forward to become a fully ready developed country, Indonesia shall move toward a more sustainable architecture for presenting a greener environment. Despite blessed with a distinctive collection of tropical material variants, reflected in its vernacular architecture, advanced material development must be invented to promote more progressive architecture in Indonesia. This research illustrates a new perspective regarding biodegradable material concepts for future Indonesian sustainable architecture. It is produced by respecting local and global development trends by using a bibliographic coupling and experimental methods in the laboratory to contribute to Indonesian sustainable architecture. A retrospective is aimed to highlight Indonesian biodegradable material and Indonesian vernacular architecture potency; it is presented as follows; (1) Understanding local–global trends in biodegradable architecture; (2) Indonesian potency on biodegradable materials; (3) A biodegradable material concept as an alternative perspective for Indonesian sustainable architecture. As a result, a new concept is proposed as an alternative for developing Indonesian biodegradable building materials. A more profound sustainable architecture is expected to engage local craftsmanship while highlighting unique biodegradable materials easily found in the surrounding environment, such as Indonesian Kombucha Tea and Indonesian Coffee.

## Introduction

The global phenomenon has been questioning the notion of sustainability towards a greener environment. Currently, the world has taken an environmental issue seriously to improve the condition. In architecture fields, the consciousness of reducing the negative effect on the intensive usage of artificial material has led to a new understanding of more sustainable architecture. The natural material is essential to be applied as the smallest unit cell of a building to construct a comprehensive sustainable architecture (Mittal and Dogne 2016). More eco-friendly material is used in construction means a greener environment is built. Although a conservative method may contribute to green design, however, to obtain the highest mark in green building certification, green label material must be included in its planning. While the green building design strategy is generally categorized as active and passive, green label material criteria are controlled by various aspects, such as location, composition, certification, transportation, and locality. The location capability to produce a green building material depends on the availability, capacity, infrastructure, craftmanship, institution. It is influenced by the relationship between the market and its customers (Greene 2019). In most developed countries, the implementation of green building design and construction is considered fundamental, as it may affect the image of a city, company branding, citizen appreciation, and other added values (Krzemińska et al. 2017). However, this implementation may be considered necessary in developing countries, including those in the transition,s budget and lower intellectual levels (Sassi 2006). Notwithstanding, Indonesia must consider the development of the green building as one of the topmost priorities for ensuring a more sustainable impact to the whole country; by producing its own local biodegradable building material followed by a green label.

**Fig. 1:**
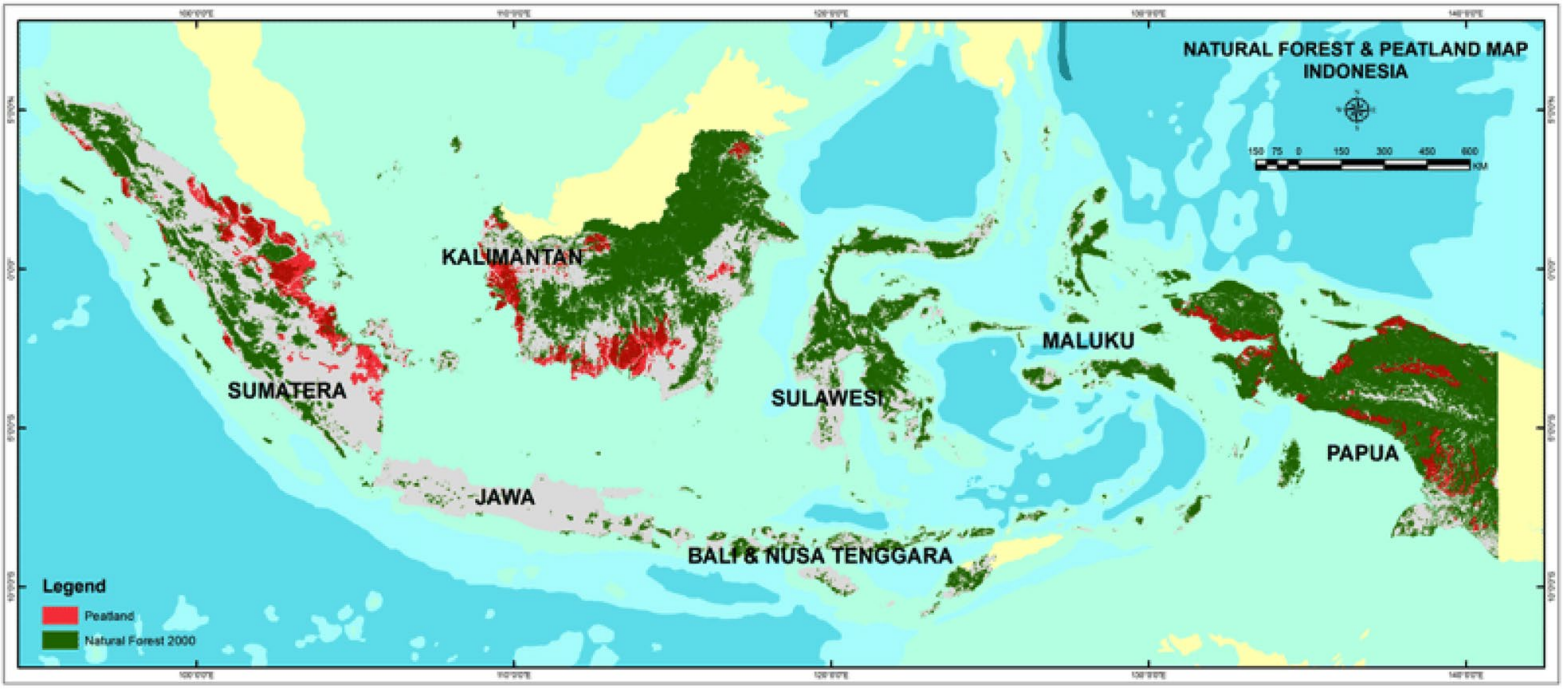
Environmental issues are illustrated by deforestation and forest degradation mapping in Indonesia. Source: BP-REDD + Indonesia, 2015 (BP-REDD+, 2015), www.reddplus.go.id downloaded on March 28, 2020

Despite recent debates that question the position of Indonesia as a developing or a developed country (Ranggasari and Bhwana 2020), in the actual situation, Indonesia is advancing towards being developed. Holding the largest economy in Southeast Asia, Indonesia is recognized as one of the megadiverse countries. Indonesia is blessed with high biodiversity that is potentially utilized as natural resources to support its economic development (UNCTAD 2019). With steadily risen GDP per capita from 2000 to 2020, Indonesia’s economic condition continues to be progressive, despite heightened global uncertainty. Nevertheless, Indonesia’s domestic demand is the main driver for its growth, as its long-term development plan is considered a challenge for Indonesia to achieve its goal. To be indeed a developed country, Indonesia must possess a fairly treated and balanced environment, including its city’s facilities and infrastructure readiness. In this sense, a progressive architecture and a more dynamic environment need to be stimulated immediately to generate advancement (Krzemińska et al. 2017). As a transcontinental country located in Southeast Asia and Oceania, the Indonesian archipelago is a valuable region for global trade. Situated strategically along the equator, Indonesia has attracted various national–international collaborators and investors targeting Indonesian bio-diversity, growing population, and rapid industrialization, despite presenting severe environmental issues (BP-REDD+ 2015), as shown by Fig. 1. Hence, to support a better economic condition, equipped with an assurance of an environmentally friendly setting, Indonesia must generate a competitive yet dynamic development, including global appreciation of green building and sustainable city. The research aims to find the concept of biodegradable materials for the future sustainable architecture of Indonesia from local natural materials typical of Indonesia. Understanding the context, following the global trend in biodegradable material while respecting local resources shall be the main agenda for developing green development in Indonesia, especially by advancing its tropical architecture.

## Material and methods

### Theoretical approach

**Fig. 2:**
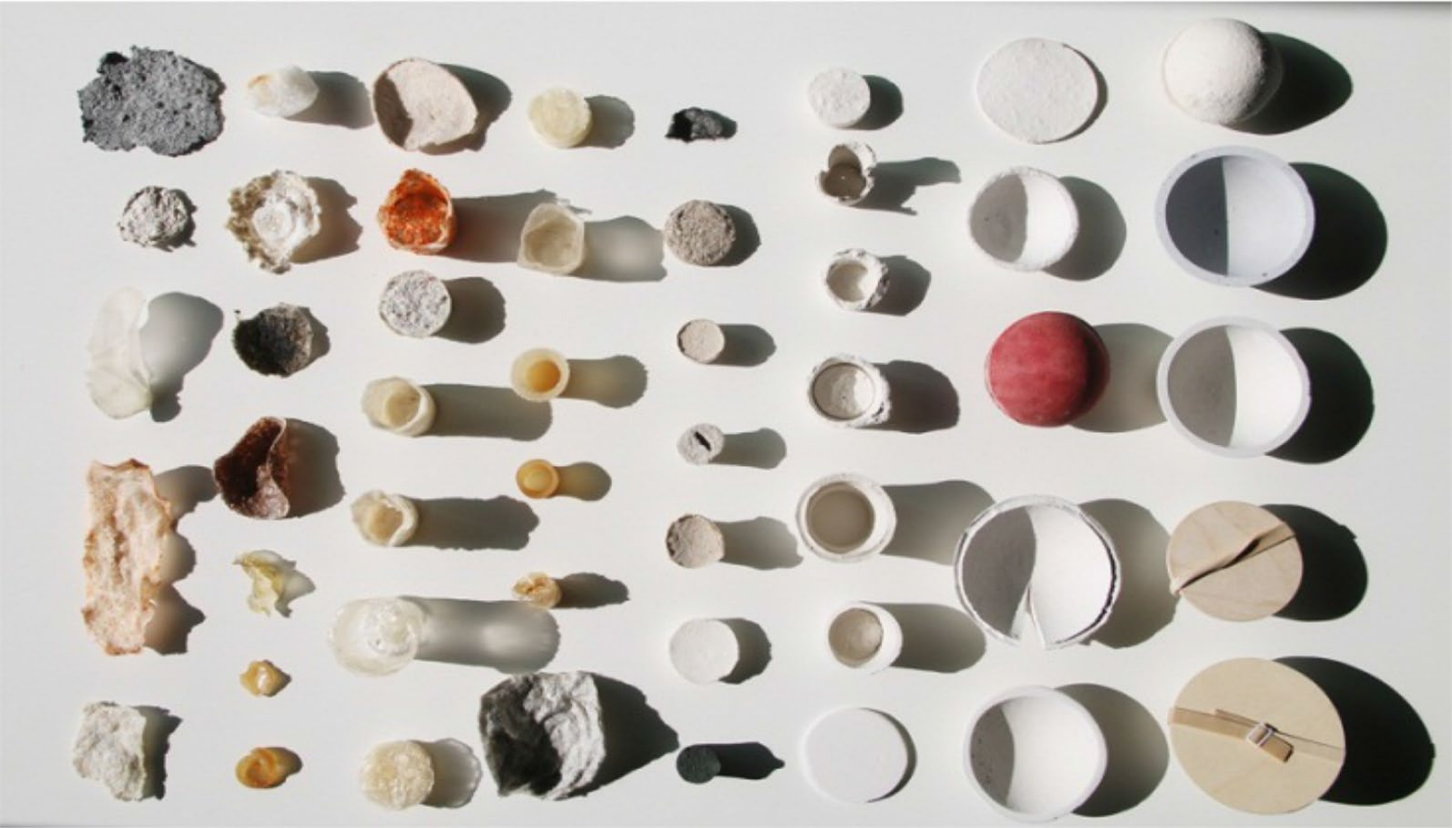
Various materials and shapes in zero experimental waste packaging by Austeja Platukyte. Source: https://www.behance.net/gallery/38533363/experimental-packaging-from-biodegradable-material downloaded on March 28, 2020

The idea of new sustainability has risen since the third millennium; the 2000 year has influenced a cultural significant around the world, contributing to the idea of a utopian future as the year has created a sense of the beginning of a new century. Climate change reports and global warming news have spread worldwide, stimulating more serious actions from reducing gas emissions to heatwaves’ control across the continents. More progressive movements are growing to shift the traditional perspective on green living towards a new sustainable generation (McLennan 2004). After two decades, the new sustainability has claimed that the future lies in regeneration. The action includes restoring ecosystems, rebalancing our climates, and rebuilding economies (Greene 2019). The landscape of innovation has set the idea of contemporary sustainability (Almy and Benedikt 2007) and work on: integrating nature with technology (Aziz and Sherif 2016), reinventing tradition for sustainability, less waste (Mostafa et al. 2018), edible products, re-use/recycle, and researching for new materials (Greene 2019). Despite varied, these actions are going towards an integrated ecosystem, respecting the process of sustainable activities, and expecting immediate results towards achieving zero waste, as illustrated by Fig. 2. When it comes to product innovation, the worlds are curious to invest in creative and scientific materials. The reborn of the renaissance is in demand; the idea of revisiting tradition, redefining classic, rethinking timeless, exploring philosophy, yet reinterpreting art bridge the gap between nature and humanity (McLennan 2004). In this sense, more designers are willing to revisit traditional materials, fusing them with dynamic new functionality (Ripley and Bhushan 2016), combining contrast ideas to create a hybrid while reducing environmental impacts (Cecchini 2017). Thus, cross-fields and multi-discipline projects offer a potency of sharing and networking, whether in inspiration, technique, and development, encouraging hybridization of biodegradable material.

Due to serious environmental issues in Indonesia, more movements are raised locally and globally to make Indonesian realize the impacts. From pollution to garbage management, Indonesia was ranked 133 out of 180 countries in 2018 according to the Environmental Performance Index (EPI 2018). This country is considered as the lowest rank amongst others located in the Asia–Pacific region. Plenty of researchers have considered Indonesia as being ignorant of the severe effects caused by irresponsible environmental activities. Therefore, more involvement and participation are stimulated to address unreduced emissions, temperature rise, and waste cycle (Mostafa et al. 2018). Architects, urban planners, developers, contractors, and designers are willing to explore new engagement; currently, it is being initiated by developing cassava plastic, bamboo textile, pineapple fibers, tapioca bags, and seaweed packaging. (Hernandha 2017). Extended research is required to perfect this biodegradable material capability to be more systematic, architectural, and degrade naturally in the environment during a time frame (Chang et al. 2017).

**Fig. 3:**
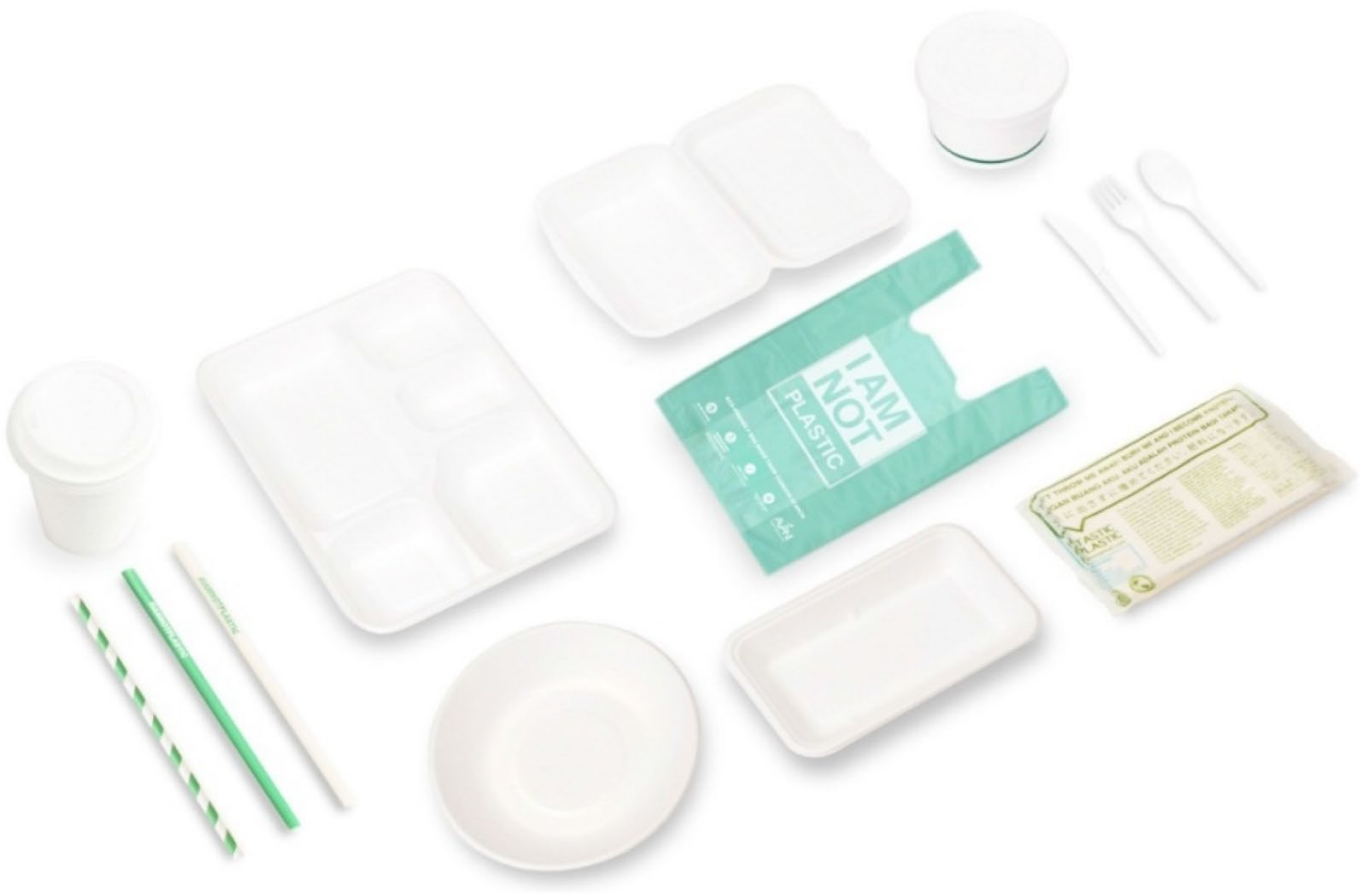
Avani Eco, a sample of the biodegradable product, produced in Indonesia. Source: https://www.avanieco.com/wp-content/uploads/2019/10/Banner-1-Replace-plastic-with.jpg, downloaded on March 28, 2020

Moreover, these varieties of biodegradable material are still only produced for smaller-scale items and targeted for selected communities, as displayed by Fig. 3. In this sense, Architecture as a medium potentially serves a bigger audience and functions for more extended periods (Krzemińska et al. 2017). Research and development of biodegradable building material may directly impact the city yet to the country (Group 2017). Nevertheless, most Indonesians still use more conventional materials and methods, driven mainly by economic aspects (Sassi 2006). Although, one must bear in mind that these users also demand to use familiar materials and methods if the new biodegradable building material is expected to hit the market successfully. Also, at the same time, advanced understanding must be improved to address the stagnancy of the design (Ripley and Bhushan 2016), as some may still worship tradition as a pure identity, a total transformation may cause a perception that the local context is being ignorant. The research shall open more opportunities towards multi-dimensional and multi-scalar products based on locality and tradition for supporting the more substantial impact while serving greater scale for achieving sustainability. A mix, combination, fusion, and other alternatives have a better potential for serving more significant communities while stimulating collaboration and networking; for example, developing food for building material, fashion architecture, edible packaging, bio-plastic for embracing synthetic yet organic materials.

Indonesia is bestowed with large wilderness areas, which means Indonesian biodiversity is considered high level: abundant with natural resources, agriculture, and various types of landscape. With a tropical climate and archipelagic geography, the country has a balanced sea, coastal ecosystems, forest, and land species distributions. Dominant in nature and culture diversity, this can be why tourism became the main attraction of Indonesia. Boast with a unique combination of a tropical climate, a vast archipelago while unified by the sea, Indonesia is bounded with a rich cultural heritage, highlighting its rich history and ethnic diversity reflected on the collection of vernacular houses. Well-known for its nature and culture globally, Indonesia is easily recognized for its traditional art and architecture as the main iconic attraction. In terms of technology development, the productivity in Indonesia is considered low (Group 2017). It is essential to inquire about the stagnancy in its cultural products (Ripley and Bhushan 2016); for example, some researchers struggle to develop freely Indonesian cultural products because the majority of Indonesian is still dictated by the notion that the traditional artifact is sacred, holy, divines, and often not advisable to be re-designed. Another case, like the traditional process, is still considered as irreplaceable, as the pure ideology is compulsory to be preserved (Hays 2015). Some intellectuals may presume this condition as stagnancy of culture, while others appreciate it as endurance of a culture. Although in the past, Indonesian is well-known for embracing various changes, transformation, and adaptation by absorbing foreign influences (Hays 2015), the same situation may not be applicable similarly at this present time. Stimulation is required to encourage a cultural hybridization by supporting elaboration, combination, and creation of biodegradable materials.

Indonesian is hugely proud of their cultures. There are some cultural products that Indonesian feels like their own identity. At the same time, they may be aware that these items may have been influenced by other cultures as a result of the acculturation process, for example, *Tempe* (soybean cake), *Tahu* (tofu), *Batik*, *Tenun* (weaving), and *Anyaman* (tapestry); and other familiar raw materials that have a strong association with Southeast Asia or archipelago, like *Kelapa* (coconut), *Rempah* (spice), *Jengkol* (dog fruit/*ngapi* nut), *Petai* (stink/bitter bean), *Durian*, *Pala* (nutmeg), and *Melati* (jasmine). These materials have latent potentials to present the identity of Indonesian culture in a glimpse, though they may not yet be familiar as a building material. Many other and prospective methods (Wahyuningtiyas and Suryanto 2017) can develop these materials into architectural products (Özdamar and Ateş 2018). Also, to encourage the application of biodegradable material, one shall revisit Indonesian vernacular architecture, which represents organic, resilient, and natural material culture (Mittal and Dogne 2016). In this sense, Indonesian architecture has a solid logical reason to reinvent advanced material, which is deeply rooted in its own culture (Gruber and Imhof 2017). By presenting the link and logic that illustrate the Indonesian-ness of a product, a guideline can be perfected to address Indonesian with full utilization of their own culture (McLennan 2004). The reason may help in constructing a sense of ownership while avoiding foreign feelings to the designed product. Although the current trend of Indonesian buildings is dominated by industrialized material, questions must be addressed to bring solutions to the homogeneity caused by modern planning. By understanding the situation, it is now our opportunity to reinvent Indonesian building material developed by crisscrossing borders between different fields (Özdamar and Ateş 2018), neglecting superficial transformation while moving forward towards global impacts (Ripley and Bhushan 2016). Hence, although traditional resources and techniques are suggested to create a familiar atmosphere for biodegradable material development in Indonesia, collaboration, proliferation, and improvisation shall stimulate innovation and creativity.

A biodegradable material concept is defined as an abstract idea or a general notion to design property of a substance that enables it to be decomposed by microorganisms (Harper 2001). The aim is to create a material that can degrade naturally in the actual environment (Wahyuningtiyas and Suryanto 2017), resulting in decay in stable conditions to reduce pollution (Sassi 2006). Although biodegradable is associated with natural matter, artificial material shall be understood as an intellectual product that can transform both raw yet waste materials into a contemporary product design (Todor et al. 2018). It shall contain calculation, strategy, and planning to adapt better to the environment while improving the way of life (Ahmed 2015). Although some industrial materials are derived from natural resources, their transformation process may not always be concerned with biodegradability. Hence, to push forward a new modern yet biodegradable building material, the industry shall be encouraged to create a more advanced natural product for building construction (Özdamar and Ateş 2018) without ignoring its biodegradability in the natural environment (Mittal and Dogne 2016). Practically, there are four standard categories of biodegradable materials, namely: minimal processing (timber, bamboo), bonded material (mixtures like carpet and board), compounds (adhesives and polymers), and synthetics (like plastics). These techniques can be implemented as building elements or components and applied during installation or construction (Sassi 2006). Thus, a biodegradable material concept in this research means a general idea to transform Indonesian natural material into building elements or components by considering its biodegradability.

**Fig. 4:**
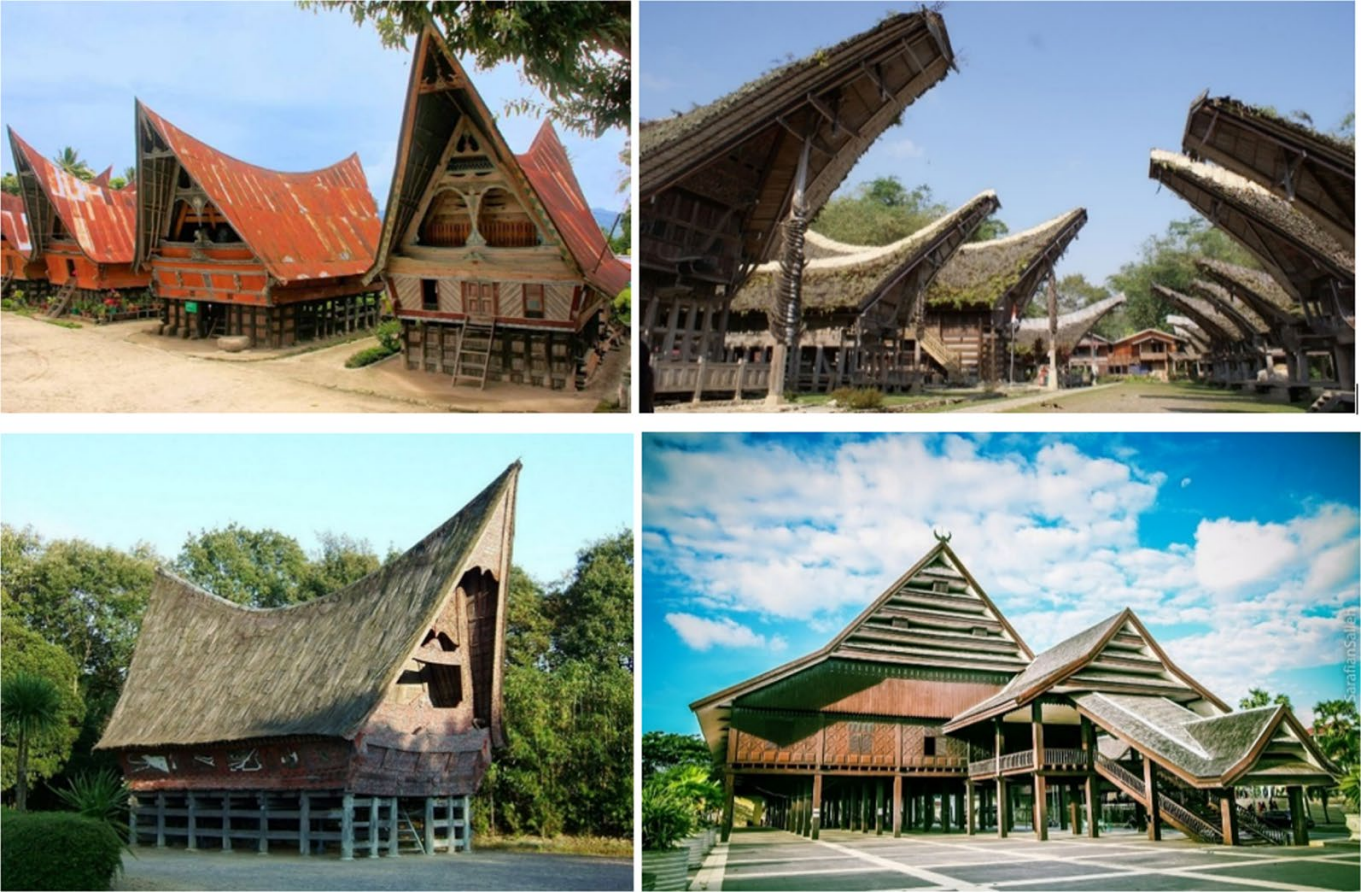
Common Natural Building Material in Indonesian Vernacular Architecture. Source: https://www.re-thinkingthefuture.com/fresh-perspectives/a1232-vernacular-architecture-of-indonesia/, downloaded on June 26, 2021

Though generic and regular material can be the resource, specific techniques and quality control must set a new standard for delivering a higher grade in biodegradable production. With generally traditional architecture and research on Indonesian natural materials are dominated respectively by (Fig. 4); (1) timber; (2) bamboo; (3) thatch; and 4) fiber; The state of the art for this research concentrates on constructing a new kind of fiber as less dominated material in Indonesian building material industry. It is created by using the edible source as a contrast to common historic building materials, which traditionally may be categorized as natural, organic, and degradable but may not yet be categorized as biodegradable according to the interpretation of the modern standard. Edible materials tend to offer a better contribution in terms of degradability, allergy reaction, safe effect, efficient production, yet consumption. Hence, by understanding the concept above, the design criteria for this research are: (1) Natural and organic; (2) Traditionally inspired; (3) Durability for 1–5 years; (4) Cross-disciplines; (5) Multi-products.

**Fig. 5:**
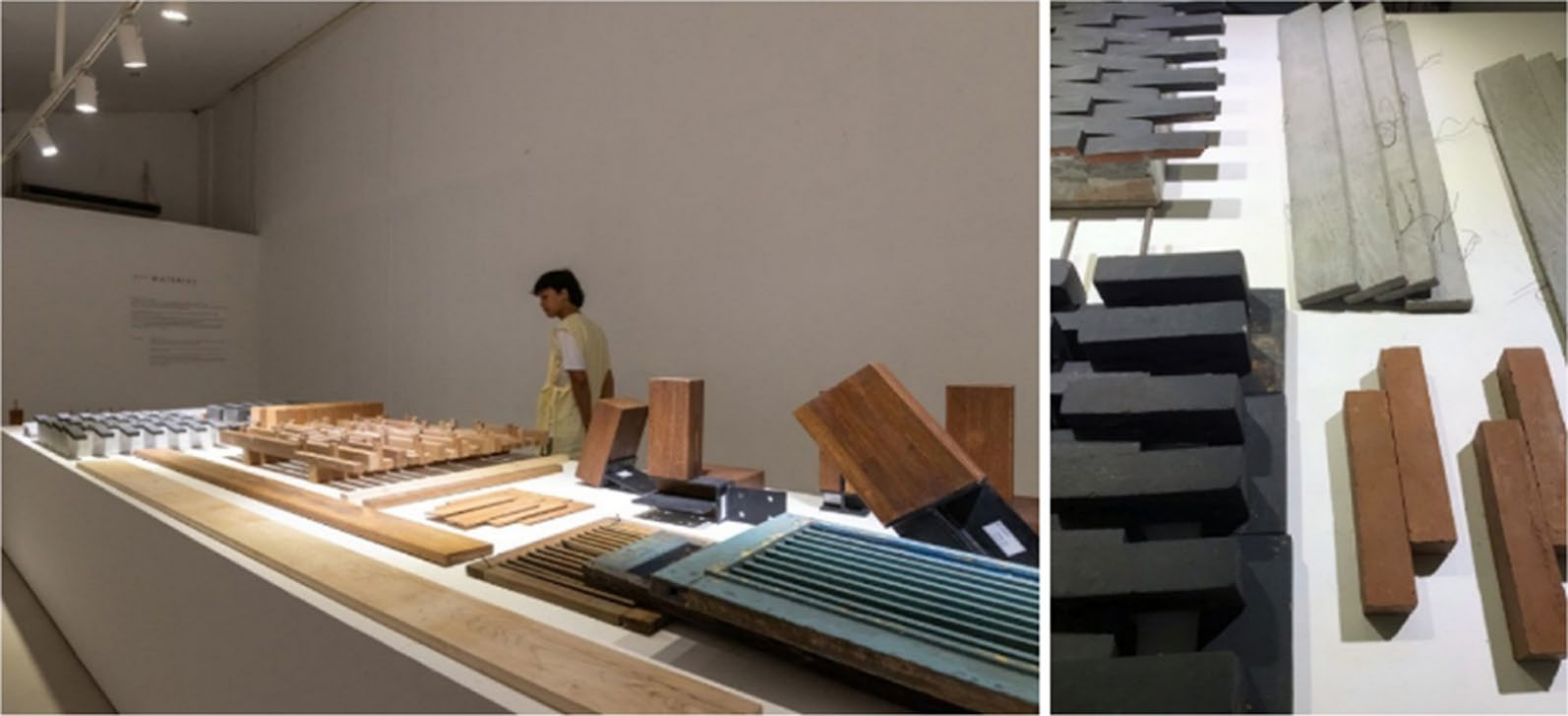
Questioning Indonesian Architecture Materials at Andra Martin *Prihal* Exhibition. Source: https://www.archify.com/id/archifynow/mengalami-arsitektur-andramatin-lewat-pameran-prihal and on March 28, 2020

The architecture of Indonesia does not only reflect its biodiversity but also representing the culture. Indigenously Indonesian architecture has been built as natural yet temporary, sot is being associated with sustainability and resilience (Mittal and Dogne 2016). Indonesian architecture illustrates the mixture of various foreign influences like Indian, Chinese, Arab, and European. In Indonesian culture, the house is the center of Indonesian customs, social relations, traditional laws, taboos, myths, and religions that bind humans with nature (Hays 2015). The earliest structures of the Indonesian vernacular house are constructed by using flexible nail-less joints and non-load bearing walls; before bricks, iron and mortar are found. In the tropical climate of Indonesia, Indonesian vernacular houses are made from traditional materials. They are produced by using various techniques like sun-drying, burned, smoked, and mostly considered hand-made. Especially in ancient times, fewer materials are initiated by neither mixture nor using machines. In the current situation, advanced technology may be found within the industry, while the practically rural community is still eager to preserve its traditional technique with minor transformation nor advanced method (Özdamar and Ateş 2018), as demonstrated by Fig. 5. Hence, there is a gap for producing a new form of biodegradable material based on Indonesian tradition, though method and technique shall be planned to match the existing craftsmanship. It delivers smooth and causing less alienation, and a new material shall be created by respecting retrospection: using Indonesian natural material executed by advanced traditional technique. Execution shall be encouraged by focusing on creating contrasts or differences, neglecting monotonous and homogeneous that is often problematic in traditional and conventional productions.

### Method of research

This research investigates a retrospective to find a biodegradable material concept for future Indonesian sustainable architecture through the bibliographic coupling method to identify the relationship between one another based on the subject’s proximity (Rupadha 2016; Glänzel and Czerwon 1996; Kessler 1963, 1965). A comparison study on local and global trends is highlighted to contrast a unique Indonesian perspective on the biodegradable material concept based on local inspiration taken from its culture and biodiversity. It is followed by experimental methods to determine the effect of specific treatments on others under controlled conditions (Sugiyono 2014; Sukardi 2011), carried out in the laboratory using natural materials suitable for producing biodegradable building materials.

The research is presented as follows; (1) Understanding local–global trends in biodegradable architecture; (2) Indonesian potency on biodegradable materials; (3) A biodegradable material concept as an alternative perspective for Indonesian sustainable architecture. A new concept is proposed as an open-ended perspective by using local materials such as Indonesian Kombucha Tea and Indonesian Coffee; it is presented as an inspiration to initiate the development of contemporary Indonesian biodegradable building material. By understanding the bigger picture, a more sustainable architecture is expected to be constructed by respecting local craftsmanship, exploring available local materials, and presenting techniques for a greener environment. Hence, this research suggests unusual inspiration for developing a concept of green building biodegradable material by using familiar Indonesian resources.

## Results and discussion

### Understanding local–global trends in biodegradable architecture

More researchers and designers around the world are currently engaged in nature-inspired objects; whether it is developed as a method, form, structure, and even function. These new items are proliferated to mimic nature to bring design closer to the actual environment. Using technology, such as 3D printing, transplantation, fermentation, and other techniques involving nature, researchers believe that parametric design, biomimicry, or genetically modified species may improve humans’ relationships and habitat. Whether producing a new generation of products, applications, or robots, various institutions are exploring more intuitive and communicative materials that interact, correspond, and decompose within their environment. Wired fabrics, compostable plastic, disposable technology are going to be trending for daily life soon. The sustainability idea in the global world now puts more concern about the overall impact that happened to the earth. A green action can now be calculated precisely and generate more participation from households to government regulation, especially becoming more common in the most developed countries. In Indonesia, the idea of sustainability is still in an initiation process, progressing towards a more advanced phase. Mostly, the initiation is an introduction of an experimental product to replace daily utensil that harms the environment. The action can be varied like replacing plastic, garbage management, and introducing re-use/recycle activity. Although there is a wide gap between local and global actions, most activities in Indonesia are inspired by international movements while finding a suitable way to be implemented in Indonesia. Indonesian are having difficulties being mindful of its richness and fortune. The act of preserving and conserving its environment is still in progress due to the overall level of education. Moreover, Indonesian rich culture and biodiversity shall not only be preserved and conserved but developed to ensure that its nature can be passed down in a more excellent way to the next generation. In this sense, regeneration must be understood as taking part in the sustainability process, elevating tradition while strengthening Indonesian cultural identity by advancing the production process.

Despite resilient, resourceful, and diverse, Indonesian architecture reflects the core of cultural values. Well-known as conservative, refined, and delicate, Indonesian must be encouraged to face the competitive world with more open-minded paradigms while preserving what they believe best. Indonesian culture emphasizes the philosophy of living in harmony, believing in subjugation to nature. However, to be progressive in sustainability, Indonesian must now be ready to question the tendency always to feel comfortable in common systems and ideology; this includes how Indonesian architecture is perceived. Despite superstitious, having a strong belief in the power of objects and events, undeniably, Indonesian has always had a solid connection to nature. It is an excellent foundation yet an open opportunity to introduce a new way of living based on essential human nature: to live harmoniously with the environment. However, to move forward, the sustainable action shall focus on an entire transformation process of developing a biodegradable material. It is best, if accompanied by easy-to-digest information and user-friendly application primarily to be delivered at home as the house is the center of Indonesian society. Biodegradable material that can be produced and applied to most houses in Indonesia means a higher level of applicability and acceptance to the broader audience. Despite Indonesia offering many natural resources that can be found throughout the archipelago, the best initiation is easily found in the surrounding living environment, whether the resource comes from the house, garden, park, and market.; whether it is a familiar building material, food ingredients, fabrics, or herbs, as long as it offers a familiarity directly to the end-user. In addition, developing natural and organic fiber at home and the surrounding environment is more visible because of its availability and affordability.

### Indonesian potency on biodegradable materials

**Fig. 6:**
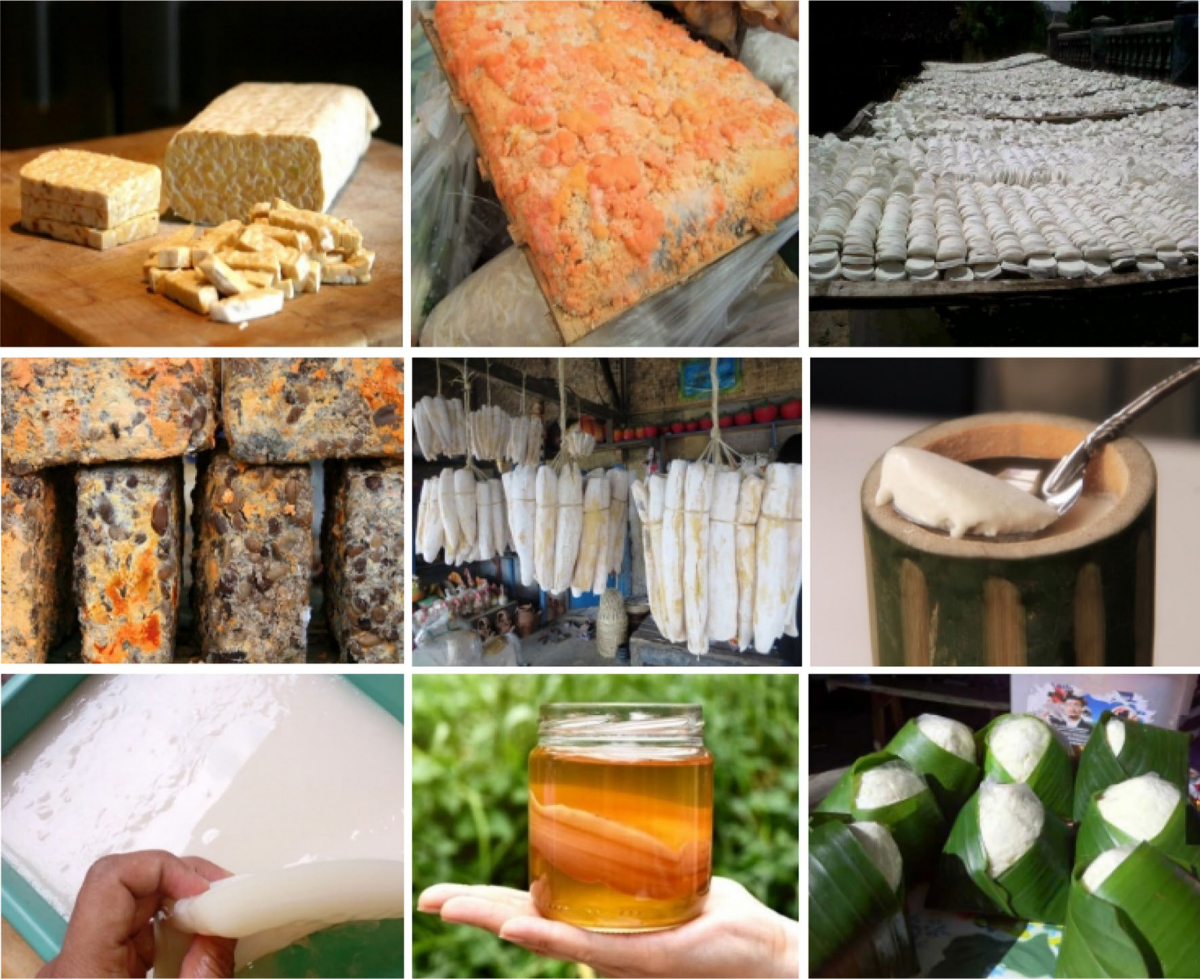
Towards Unexplored Potency of Indonesian Biodegradable Material; Left to Right: Soybean cake, Red soy oil-cake, Solid fermented glutinous rice, Black soy oil-cake, Fermented cassava, Buffalo milk yogurt, Nata de coco, Kombucha, *Enrekang* cheese. Source: https://www.indoindians.com/fermented-foods-from-indonesia/. https://4.bp.blogspot.com/-QEqSTFN53Vo/WlGzolFTJkI/AAAAAAAABKI/K05RdGnvBGknCZ8gulJSZjTYy5jhAncCQCLcBGAs/s1600/IMG_20170721_143344.jpg. https://storage.trubus.id/storage/app/public/posts/t20190925/big_39475912023d773c4b210e2062cd32a5fe904bd0.jpg downloaded on March 28, 2020

According to the stagnancy of technology and application of homogeneous material in the existing setting; a biodegradable material concept as an alternative for Indonesian sustainable architecture must highlight the importance of a new method, advancing the final product for moving from only preserving and conserving a raw traditional building material to the advanced ones. A superficial transformation shall be shifted to the exploration of complex material and technique. This action needs to completely change the overall formation, structure, function, or even genetics of the designed materials. Crosschecking with the latest global trends: fish scale, skin waste, crustacean exoskeleton, eggshell, algae, ground coffee beans, rice husk, fruit peel, corn husks, wool, yogurt pots, tree cellulose, fungus, yeast, fermented farm waste, food waste, animal skin, sunflower seeds also bee wax can be developed as biodegradable materials. It is almost every natural thing that can be converted and transformed into biodegradable building material. It means Indonesian exploration of biodegradable does not need to be afraid to explore different bio things, moving forward from one or two popular material choices as in the current situation is dominated by limited types. Also, the exploration may be continued by the advancement of different techniques like fermentation, yeast, distillation, brew, inspired by various traditional methods rather than repeating common methods, as exemplified by Fig. 6. Although these traditional methods are common in Indonesia, especially for the home industry, implementation is relatively stagnant or steady than embracing diversity. Exploration shall be encouraged to demonstrate different, contrast, distinction, and divergence in terms of color, texture, size, the process even performance for achieving the advancements.

However, to represent the Indonesian-ness, one shall choose a natural resource or method locally and globally recognized as Indonesian, although a total rework needs to be planned to advance the invention. Hence, in this sense, the research highlights the unlimited potentials of Indonesian biodiversity as the concept for biodegradable architecture material to address the stagnant inspiration. Also, to address current problems on developing global biodegradable material, the following Indonesian projects need to be perfected by ensuring its capability to decay smoothly to the actual environment. Also, the research shall be extended to be more architecture as the option for biodegradable building material is still considered scarce. Simulation, calculation, and prediction are needed to be encouraged to generate a greener biodegradable material, presenting a better future for a more sustainable environment. Despite unlimited inspiration that can be found from Indonesian resources, the problem remains the same and is dominated by orthodoxies. The gap and the real challenge are where the Indonesian-ness must be interpreted as the new output.

### A biodegradable material concept as an alternative perspective for Indonesian sustainable architecture

Two inspirations are selected for experimenting with traditional materials and techniques. Elaborations and proliferation are considered for presenting various colors, textures, sizes, and characters by using a variety of types, concentrations, intensification. However, the resource of material and techniques remain conventional. This home experimentation leads to producing different biodegradable materials related to green buildings and is chosen because of the availability and affordability of Indonesia’s general public. Two familiar materials have been investigated for 2 years in this experimentation; tea and coffee as the main ingredients. The first one is intended for creating sheets, and the other as insulation or filler. First initiation using kombucha tea generally leads to vegan leather, *fusuma*, wallpaper sheet, bioplastic, and translucent materials. A different utilization of tea, sugar, water may produce a natural variation of color, odor, texture, and thickness that contribute to different qualities and quantities of biomaterials. Various agents like natural fat, oil, and butter can be added to enhance the products’ flexibility and elasticity. Preservation techniques like smoking, frying, sundry stimulates diversity in terms of finishes and level of conservation. The second experimentation uses coffee waste. This experiment leads to the creation of material boards, bio-wood, insulations, and insect repellent. Different natural materials like tapioca, cornstarch, chalk, salt, and agar–agar are tested as variations for stabilizers and additional agents. Elaboration is done by fermentation, drying, frying, and molding (Table 1). This home experimentation leads to generally stable material for up to 6 months to 1 year before degrading smoothly at the end of the year; notwithstanding, both materials are generally water-soluble because of their organicity. Scientific and lab experiments must establish a higher quality standard material and push for more extended durability.

**Table 1:**
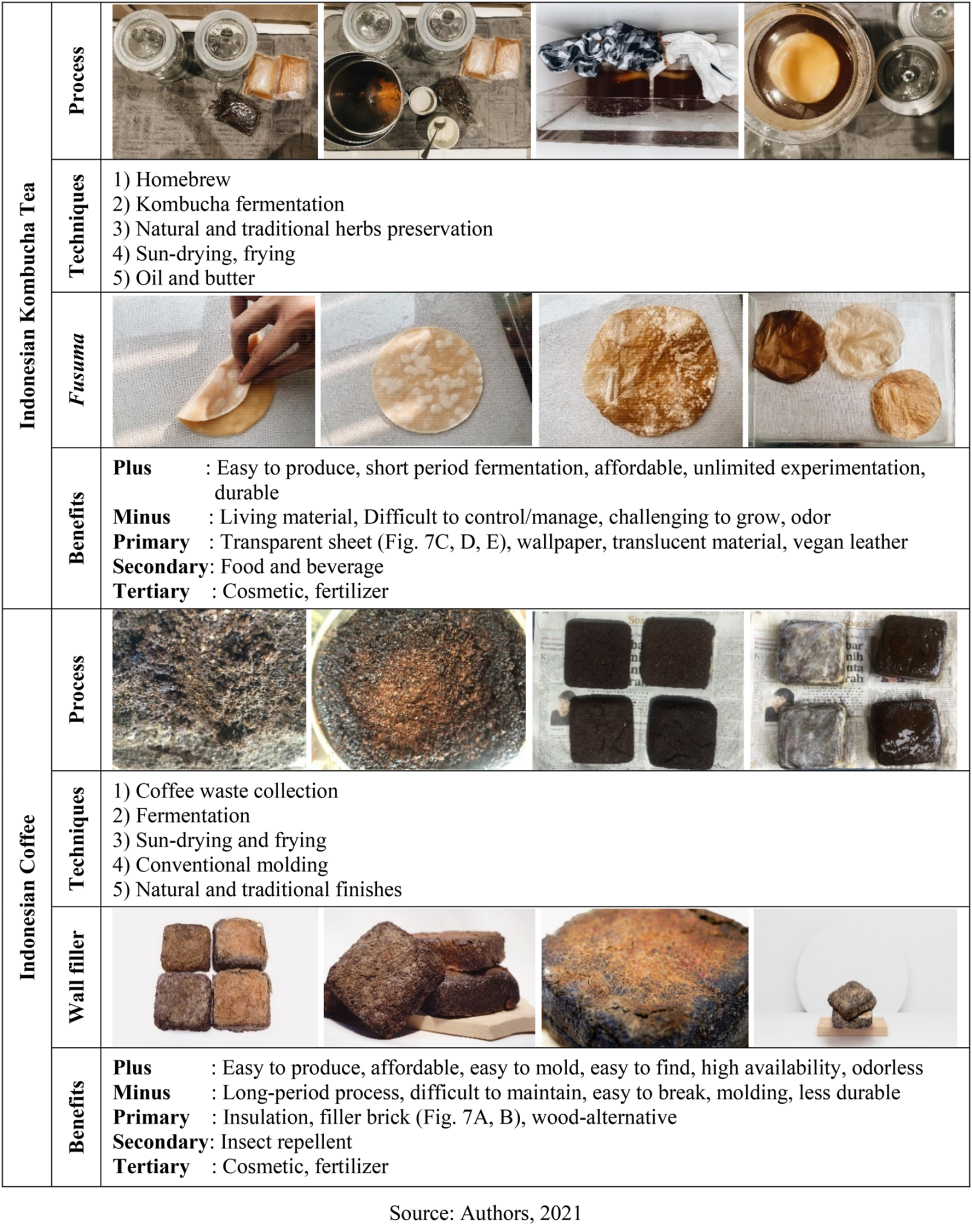
Experimentation on Kombucha tea and coffee waste for biodegradable material concept Source: Authors, 2021f

**Fig. 7:**
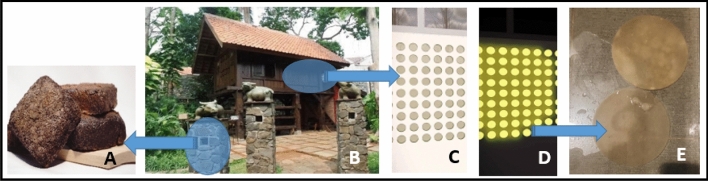
Illustration of the use of Coffee Waste Materials (**A**) and Kombucha Tea (**E**); Eksterior view (**C**) and Interior view (**D**) of Kombucha Tea Applications. Source: Elaborated Photo (**B**) from *Penerapan Arsitektur Neo–Vernakular pada Bangunan Fasilitas Budaya dan Hiburan* (Widi and Prayogi 2020)

Illustrations of applying the experimental results above: (1) Indonesian coffee waste as filler wall bricks or decorative pillars (Fig. 7A, B); (2) Indonesian Kombucha tea as a semi-translucent material so that sunlight enters the room, the use of colorful kombucha tea gives a stunning impression (artistic) on the interior and appearance of the building (Fig. 7C, D, E).

## Conclusions

As a country in a transitional economy, Indonesia requires more effort to make its environment ready for global competition. It includes its architecture and city to prepare for the international standard to accommodate global networks and activities. Although undeniably Indonesia is rich with biodiversity and various inspirations to develop a biodegradable building material, active participation and support from various sectors are needed to open up the possibility for creating a greener environment. As Indonesia is in the initiation phase of introducing biodegradable material to its society, an understanding needs to be lifted to the broader audience to anticipate homogeneous interpretations and creating a multi-dimensional perspective.

There are three highlights for developing the general idea of the biodegradable material concept for future Indonesian sustainable architecture; (1) There is a local stagnancy of interpreting inspiration for biodegradable building material. It is often taken concerning Indonesian neo-vernacular architecture, but often seen as a limitation and barrier, while the global trend has clarified the unlimited possibility of designing biodegradable material almost from anything, including reusing and recycling ‘artificial/synthetic’ materials; (2) Indonesia can produce various biodegradable material developed from diverse materials, including the one may not be presented yet in Indonesian neo-vernacular architecture. Hence, although inspiration and lessons are taken from local architecture and traditional material, it is essential to propose a unique biomaterial product to contrast what is provided by existing industry.

Material sources found in the surrounding environment should be prioritized. The material and technique shall be developed at home and able to be done by engaging local craftsmanship. Thus, alienation may be avoided. It may be contrasted with the case in other developed societies where an application of unfamiliar sources of biodegradable material to a public building is intended as part of the attraction, promotion, and education for the general public; (3) The future of Indonesian biodegradable material is omnipresent. However, to encourage sustainable architecture, Indonesia shall move from only implementing raw material and conservative methods to encouraging material development from the minor structure. Advanced techniques are suggested to stimulate innovation, for example, biomolecular engineering, biotechnology, and biochemistry; thus, multi-discipline collaboration can be promoted. The production must present a calculated, predicted, and simulated complex process of fermentation, culture lab, brewing, distillery, genetically modified products to present a more advanced biodegradable building material for future Indonesian sustainable architecture, such as Indonesian Kombucha Tea and Indonesian Coffee. Hence, lab experimentation and scientific methods are required for presenting higher quality material and universal design.

## Data Availability

Not applicable.

## References

[CR1] Ahmed MM (2015). Bio-Digital Morphogenesis in Architecture: An Application on Digital—Botanic Architecture. Thesis Graduate School Faculty of Engineering, Alexandria University. Alexandria, Egypt: Alexandria University. https://www.cpas-egypt.com/pdf/Mahmoud_Mohamed_Gomaa/MS.c.pdf. Accessed 15 May 2020

[CR2] Almy D, Benedikt M (2007). Center 14: on landscape urbanism.

[CR3] Aziz MS, Sherif AY (2016). Biomimicry as an approach for bio-inspired structure with the aid of computation. Alex Eng J.

[CR4] BP-REDD+ (2015) National Forest Reference Emission Level for Deforestation and Forest Degradation in the Context of the Activities Referred to in Decision 1/CP.16. Jakarta: BP-REDD+ Indonesia

[CR5] Cecchini C (2017). Bioplastics made from upcycled food waste. Prospects for their use in the field of design. Des J.

[CR6] Chang TJ, Yao Z, Jackson PJ, Rand BP, Wentzlaff D (2017) Architectural Tradeoffs for Biodegradable Computing. MICRO-50'17. In: Proceedings of the 50th Annual IEEE/ACM International Symposium on Microarchitecture. Cambridge. MICRO-50, pp 706–717. 10.1145/3123939.3123980

[CR7] EPI (2018) https://epi.envirocenter.yale.edu/downloads/epi2018policymakerssummaryv01.pdf. https://epi.envirocenter.yale.edu/. https://epi.envirocenter.yale.edu/downloads/epi2018policymakerssummaryv01.pdf. Accessed 28 Mar 2020

[CR8] Glänzel W, Czerwon HJ (1996). A new methodological approach to bibliographic coupling and its application to the national, regional and institutional level. Scientometrics.

[CR9] Greene L (2019) The Future 100. New York: Innovation Group: J. Walter Thompson Intelligence. https://www.thegeniusworks.com/wp-content/uploads/2018/11/Future-100_2019.pdf. Accessed 26 May 2020

[CR10] Gruber P, Imhof B (2017). Patterns of growth—biomimetics and architectural design. Buildings.

[CR11] Harper D (2001) Online etymology dictionary biodegradable definition. https://www.etymonline.com. https://www.etymonline.com/word/biodegradable. Accessed 25 July 2019

[CR12] Hays J (2015) http://factsanddetails.com/indonesia/People_and_Life/sub6_2a/entry-3987.html. http://factsanddetails.com/. http://factsanddetails.com/indonesia/People_and_Life/sub6_2a/entry-3987.html. Accessed 28 Mar 2020

[CR13] Hernandha RF (2017) https://www.goodnewsfromindonesia.id/2017/11/23/indonesia-siap-melawan-plastik-non-biodegradable-dengan-singkong-dan-rumput-laut*.* https://www.goodnewsfromindonesia.id/. https://www.goodnewsfromindonesia.id/2017/11/23/indonesia-siap-melawan-plastik-non-biodegradable-dengan-singkong-dan-rumput-laut. Accessed 28 Mar 2020

[CR14] Kessler M (1963). Bibliographic coupling between scientific papers. Am Doc.

[CR15] Kessler M (1965). Comparison of the results of bibliographic coupling and analytic subject indexing. Am Doc.

[CR16] Krzemińska A, Zaręb A, Dzikowska A (2017). Bioarchitecture—a new vision of energy sustainable cities. Int Conf Adv Energy Syst c Environ Eng..

[CR17] McLennan JF (2004). The philosophy of sustainable design.

[CR18] Mittal G, Dogne N (2016) Sustainable Architecture in Terms of Building Materials. J Civ Constr Eng. 2(1):1–7. https://www.academia.edu/32481868/Sustainable_Architecture_in_Terms_of_Building_Materials. Accessed 27 June 2020

[CR19] Mostafa N, Farag A, Abo-dief H, Tayeb A (2018). Production of biodegradable plastic from agricultural wastes. Arab J Chem.

[CR20] Group OB (2017) https://oxfordbusinessgroup.com/news/indonesia-seeking-greater-funding-rd. https://oxfordbusinessgroup.com/: https://oxfordbusinessgroup.com/news/indonesia-seeking-greater-funding-rd. Accessed 28 Mar 2020

[CR21] Özdamar E, Ateş M (2018). Rethinking sustainability: a research on starch based bioplastic. J Sustain Constr Mater Technol.

[CR22] Ranggasari R, Bhwana P (2020) https://en.tempo.co/read/1311451/us-removes-indonesia-from-developing-countries-list. https://en.tempo.co/. https://en.tempo.co/read/1311451/us-removes-indonesia-from-developing-countries-list. Accessed 28 Mar 2020

[CR23] Ripley RL, Bhushan B (2016). Bioarchitecture: bioinspired art and architecture—a perspective. Philos Trans Roy Soc A Math Phys Eng Sci.

[CR24] Rupadha IK (2016). Memahami Metode Analisis Pasangan Bibliofragi (Bibliographic Coupling) dan Ko-Sitasi (Co-Sitation) serta Manfaatnya untuk Penelitian Kepustakaan. Lentera Pustaka.

[CR25] Sassi P, Brebbia CA (2006). Biodegradable building. Design and nature III: comparing design in nature with science and engineering.

[CR26] Sugiyono (2014). Metode Penelitian Kuantitatif, Kualitatif dan R&D.

[CR27] Sukardi (2011). Metodologi Penelitian Pendidikan Kompetensi dan Praktiknya.

[CR28] Todor M, Bulei C, Heput T, Kiss I (2018) Researches on the development of new composite materials complete/partially biodegradable using natural textile fibers of new vegetable origin and those recovered from textile waste. In: International Conference on Applied Sciences (ICAS2017); IOP Conf. Series: Materials Science and Engineering, vol 294. IOP Publishing, pp 1–9. 10.1088/1757-899X/294/1/012021

[CR29] UNCTAD. (2019). https://unctad.org/en/Pages/DITC/Trade-and-Environment/BioTrade/BT-Indonesia.aspx. https://unctad.org/. https://unctad.org/en/Pages/DITC/Trade-and-Environment/BioTrade/BT-Indonesia.aspx. Accessed 28 Mar 2020

[CR30] Wahyuningtiyas NE, Suryanto H (2017) Analysis of biodegradation of bioplastics made of cassava starch. J Mech Eng Sci Tech 1(1):24–31. http://journal2.um.ac.id/index.php/jmest/article/view/1207/0

[CR31] Widi CD, Prayogi L (2020). Penerapan Arsitektur Neo–Vernakular pada Bangunan Fasilitas Budaya dan Hiburan. J Arsit ZONASI.

